# Optimising preoperative risk stratification tools for prostate cancer using mpMRI

**DOI:** 10.1007/s00330-017-5031-5

**Published:** 2017-10-06

**Authors:** Lars A. R. Reisæter, Jurgen J. Fütterer, Are Losnegård, Yngve Nygård, Jan Monssen, Karsten Gravdal, Ole J. Halvorsen, Lars A. Akslen, Martin Biermann, Svein Haukaas, Jarle Rørvik, Christian Beisland

**Affiliations:** 1 Department of Radiology, Haukeland University Hospital, Jonas Liesvei, N-5021 Bergen, Norway; 2 Department of Clinical Medicine, University of Bergen, Bergen, Norway; 3 Department of Radiology, Radboud University Nijmegen Medical Centre, Nijmegen, The Netherlands; 4 Department of Urology, Haukeland University Hospital, N-5021 Bergen, Norway; 5 Department of Pathology, Haukeland University Hospital, N-5021 Bergen, Norway; 6 Centre for Cancer Biomarkers CCBIO, Department of Clinical Medicine, University of Bergen, Bergen, Norway

**Keywords:** Prostate cancer, Biochemical recurrence, Risk stratification, Prostate mpMRI, MRI

## Abstract

**Purpose:**

To improve preoperative risk stratification for prostate cancer (PCa) by incorporating multiparametric MRI (mpMRI) features into risk stratification tools for PCa, CAPRA and D’Amico.

**Methods:**

807 consecutive patients operated on by robot-assisted radical prostatectomy at our institution during the period 2010–2015 were followed to identify biochemical recurrence (BCR). 591 patients were eligible for final analysis. We employed stepwise backward likelihood methodology and penalised Cox cross-validation to identify the most significant predictors of BCR including mpMRI features. mpMRI features were then integrated into image-adjusted (IA) risk prediction models and the two risk prediction tools were then evaluated both with and without image adjustment using receiver operating characteristics, survival and decision curve analyses.

**Results:**

37 patients suffered BCR. Apparent diffusion coefficient (ADC) and radiological extraprostatic extension (rEPE) from mpMRI were both significant predictors of BCR. Both IA prediction models reallocated more than 20% of intermediate-risk patients to the low-risk group, reducing their estimated cumulative BCR risk from approximately 5% to 1.1%. Both IA models showed improved prognostic performance with a better separation of the survival curves.

**Conclusion:**

Integrating ADC and rEPE from mpMRI of the prostate into risk stratification tools improves preoperative risk estimation for BCR.

***Key points*:**

• *MRI-derived features, ADC and EPE, improve risk stratification of biochemical recurrence.*

• *Using mpMRI to stratify prostate cancer patients improves the differentiation between risk groups.*

• *Using preoperative mpMRI will help urologists in selecting the most appropriate treatment.*

**Electronic supplementary material:**

The online version of this article (10.1007/s00330-017-5031-5) contains supplementary material, which is available to authorized users.

## Introduction

Prostate cancer (PCa) is the most common non-cutaneous malignancy affecting men in developed countries. The recommended treatment for highly selected low-risk disease is active surveillance rather than radical therapy such as radical prostatectomy (RP) or external beam radiation therapy [1]. A major challenge in treating PCa is to identify all patients with intermediate- and high-risk disease who need radical treatment while avoiding overtreatment in the low-risk group.

Biochemical recurrence (BCR) after RP is a well-established predictor for clinical recurrence and disease-related mortality. Two-thirds of BCR occur early, within 2 years of RP [2]. BCR after RP is defined as an elevated serum prostate-specific antigen (s-PSA) above 0.2 ng/ml after nadir 4–6 weeks after surgery [3]. BCR is highly dependent on the stage of the disease, as well as on the level of surgical performance. Pavlovich et al. found BCR rates of 1.8% for pT2N0/Nx and 22.3% for pT3N0/Nx/N1 [4]. The cohort-dependent differences in BCR rates led to a proposal for risk-adjusted follow-up based on three risk levels of BCR: low, intermediate and high [5].

Positive surgical margins (SM+), extraprostatic extension (EPE), biopsy and postoperative Gleason scores have all been reported as reliable predictors of BCR [6, 7]. Risk stratification tools, such as the University of California San Francisco Cancer of the Prostate Risk Assessment tool (CAPRA) and D’Amico, are routinely used to advise patients for or against radical therapy including surgery on the basis of preoperative information. D’Amico is based on s-PSA, biopsy Gleason score and clinical T stage on digital rectal examination (DRE) [8], while CAPRA additionally takes into account patient age and the percentage of positive biopsy cores [9] and is validated for European patients [10, 11].

During the last decade, multiparametric MRI (mpMRI) of the prostate was introduced for detection and localisation of PCa. Recent studies indicate that mpMRI has a high diagnostic accuracy for detection of PCa [12, 13], can improve predictions of preoperative clinical nomograms, at least for locally advanced disease [14, 15], and that mpMRI may both improve predictions of clinical BCR nomograms [16–19] and improve risk stratification for detection of significant PCa [20].

We aimed to improve the prognostic accuracy of the two most widely used clinical prediction tools, CAPRA and D’Amico, by including pertinent mpMRI features such as apparent diffusion coefficient (ADC), radiological EPE (rEPE) and tumour size into new, image-adjusted (IA) prediction models.

## Materials and methods

### Patients

**Fig. 1:**
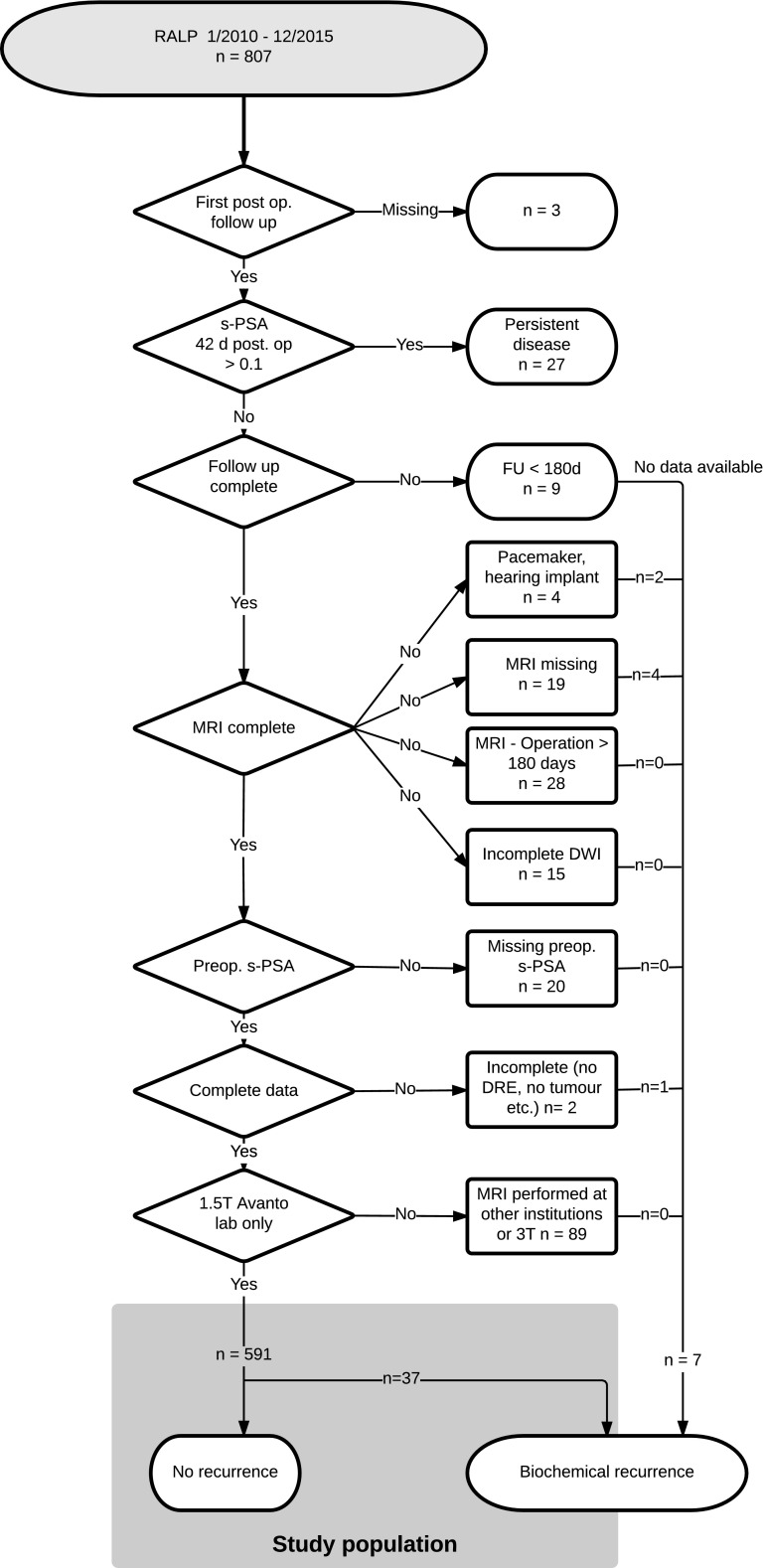
STARD diagram detailing the stepwise exclusion leading to the study cohort

The study population comprised 807 prospectively enrolled consecutive patients that underwent robot-assisted laparoscopic radical prostatectomy (RALP) for PCa at our institution between 1 January 2010 and 31 December 2015. Patients with persistent elevated s-PSA after RALP, follow-up of less than 180 days, MRI examination performed at 3 T or at other institutions, or missing observations were excluded from analysis (Fig. 1). A total of 591 patients were eligible for analysis, of which 59 received adjuvant radiation therapy. Preoperative s-PSA measurements were all taken within 3 days prior to the operation. s-PSA levels before the operation and at nadir 4–6 weeks after RALP were all analysed at the same laboratory between 1 January 2010 and 30 June 2016. The institutional review board approved this study, and all patients gave their written consent.

### MRI protocol

Eighty-seven per cent (591/680) of all patients with preoperative mpMRI were examined using the same 1.5-T MR machine (Avanto; Siemens Medical Systems, Erlangen, Germany) and scanning protocol (Table 1).

**Table 1:** Scanning protocol

Sequence	Plane	Repetition time/echo time (ms)	Intersection gap (mm)	Matrix	Field of view (mm)	Acquisition time
T2W	Sag	3030/98	0.8	320 × 256	200 × 200	3:06 min
T2W	Cor	3000/98	0.4	320 × 256	200 × 200	4:05 min
T2W	Axial	4840/84	0.8	320 × 256	200 × 200	4:18 min
VIBE	Axial	7.23/2.55	0.8	192 × 192	250 × 250	20 s
DWI (b50, 400, 800)	Axial	3000/72	0.8	128 × 128	128 × 128	5:33 min
DWI (b1200)	Axial	2800/83	0.6	128 × 128	250 × 250	2:23 min
DCE TWIST + C	Axial	4.24/1.66	0.8	512 × 512	192 × 138	6:58 min

*Sag* sagittal, *Cor* coronal, *T2W* T2-weighted imaging, *T1* T1-weighted imaging, *VIBE* volumetric interpolated breath-hold examination, *DWI* diffusion-weighted images, *DCE TWIST* dynamic contrast-enhanced time-resolved interleaved stochastic trajectories sequence, *iPAT 2* time resolution = 6.16 s, *+ C* with intravenous contrast

### MRI interpretation

All mpMRI examinations of the prostate were retrospectively read in random order by one radiologist (LR) blinded to clinical information and outcomes, except that the patients had been allocated to RP for PCa. The radiologist had more than 10 years’ experience of reading prostate mpMRI, presently interpreting at least 800 examinations per year. rEPE was assessed on the basis of the criteria bulging, asymmetric capsule and capsule contour deviations [21] using a Likert scale from 1 to 5, where 1 = no presence of EPE, 2 = probably no presence of EPE, 3 = uncertain of the presence of EPE, 4 = EPE probably present, 5 = EPE definitely present. We threshold the scores at 4 or above indicating the presence of EPE. Receiver operating characteristics (ROC) for rEPE are shown in supplementary Fig. S1.

### Image processing

The radiologist identified the leading lesion with the highest Prostate Imaging Reporting and Data System (PI-RADS) score by using PI-RADSv1 (10) blinded to final histopathology result. The lesion size was measured on axial T2W images as well as the ADC value from a region of interest (ROI), including 2/3 of the lesion on axial ADC maps. The radiologist did not use software for segmentation or sophisticated processing tools.

### Surgery

RALP was introduced as standard surgical treatment for PCa at our institution in May 2009. Four surgeons performed all the procedures. Extended lymph node dissection [22] was performed in 357 of the 807 patients (44%) during the study period.

### Histopathology

Whole-mount step sections were taken from the prostatectomy specimens at 5-mm intervals. The pathologists outlined the presence and extent of tumour involvement in drawings based on the histological sections of the entire prostate and determined the presence of a pathologic index tumour based on the International Society of Urological Pathology (ISUP) consensus conference criteria [23].

The volume of the tumours was estimated using routine pathologic measurements as previously described [24]. The presence of EPE and SM+ was noted in each patient. In addition, all preoperative biopsy data that are part of the CAPRA and D’Amico tools were included in the database, including Gleason grade and score and the percentage of positive biopsy cores. In accordance with the 2014 ISUP consensus, Gleason grade scores were grouped into five grade groups [25], grade groups being both simpler and more robust [26].

### Data and statistics

All data were collected in a custom-developed relational database that maintained blinding of the independent observers [27]. Observations from individual tables were re-aggregated using Structure Query Language views. Statistical analyses were then performed using R 3.3.1 [28] utilising the packages EpiR, survival, ROCR, DecisionCurve and hdnom. Continuous variables were summarised by median and mean values, and interquartile ranges. To evaluate predictors of early BCR, we used Cox hazards backward stepwise likelihood ratio methodology with the lowest Akaike information criterion (AIC) [29]. In addition, we applied a penalised Cox regression model with leave-one-out cross-validation (LOOCV), using an elastic net to estimate the linear effect of the predictors, optimised at the simplest model that has comparable error (1 standard deviation) to the best model given the uncertainty [30]. To compare the performance of the prediction tools we used ROC and DeLong’s test, survival analysis, decision curve analysis and ANOVA. All statistical tests were applied at a significance level of 5% (two-sided).

### Creating the IA model

The pre-existing risk groups defined by CAPRA/D’Amico were assigned 1 point for low risk, 2 points for intermediate risk and 3 points for high risk. Stepwise backward likelihood ratio testing and LOOCV identified two common mpMRI features, statistically significant by LOOCV, namely ADC and rEPE. To weight the mpMRI features, we ran an LOOCV analysis based on the full preoperative and postoperative variables, creating the nomogram shown in supplementary Fig. S2. In this nomogram, an ADC value less than 800 mm^2^/s added 55 points to overall risk while rEPE+ equated to 60 points. We decided to weight points for ADC measurement into four groups, based on the upper and lower boundaries of interquartile range at 650 mm^2^/s and 961 mm^2^/s of our cohort. We thus chose a cut-off value of ADC at 800 mm^2^/s, being almost 150 mm^2^/s from each interquartile range. For simplicity, we chose ADC value cut-off points at 650, 800 and 950 mm^2^/s, resulting in groups of almost equal size.

For our IA model, we therefore assigned 0 points for ADC values at the level of upper quartile around greater than 950 mm^2^/s, + 1 point for 950–800 mm^2^/s and + 2 points for 799–650 mm^2^/s, and finally + 3 points for ADC values below the lower quartile at approximately less than 650 mm^2^/s. If rEPE was present (score ≥ 4), 2 points were assigned; if rEPE was not present (score ≤ 3), 0 points were assigned. The points were added to the pre-existing risk group points (low = 1, intermediate = 2, high = 3) assigned by CAPRA/D’Amico. This results in an eight-point risk scale with 1–2 = low risk, 3–5 = intermediate risk, 6–8 = high risk.

## Results

Of the 591 patients included in the final analysis, 37 (6.3%) were identified with early BCR. Surgical margins were positive in 90 patients (15.2%); 49 of these were pT3 (33.8% of all pT3), while 41 were pT2 (9.2% of all pT2). The median length of SM+ for pT2 and pT3 was 2.5 mm and 2.7 mm, respectively. Eighteen patients (3%) had N+ disease and 51 (8.6%) patients received adjuvant therapy. Additional patient characteristics are listed in Table 2.

**Table 2:** Patient characteristics *n* = 591

Biopsy age (years)	Median (IQ)	63.3 (59.4–66.1)	D’Amico	Low	126
				Intermediate	370
cT stage (DRE)	T1c	427		High	95
	T2a	91			
	T2b	45	CAPRA score	Low	139
	T2c	17		Intermediate	358
	T3a/b	11		High	94
Grade group (biopsy)	GG 1	195			
	GG 2	256			
	GG 3	81			
	GG 4	47			
	GG 5	12			
# Positive biopsy cores (%)	Median (IQ)	36 (21–50)			
mpMRI to RALP (days)	Median (IQ)	28 (14–62)	Size T2W (mm)	Median (IQ)	18 (13–23)
MRI indicating EPE, Likert score ≥ 3	rEPE–	377	Size ADC (mm)	Median (IQ)	17 (12–24)
	rEPE+	214 (36%)			
Lowest ADC-value in visible tumour	Median (IQ)	767 (650–961)			
Operation age (years)	Median (IQ)	63.6 (59.6–66.5)	Grade group	GG 1	78
				GG 2	357
Operation s-PSA (ng/ml)	Median (IQ)	8.4 (6.1–12.0)		GG 3	113
				GG 4	12
				GG 5	31
pT stage (‘92 classification)	T2a	27			
	T2b	24		(1)	No tumour
	T2c	395			
	T3a	104			
	T3b	15			
	T3c	26			
EPE	Not present	493			
	Present	98 (16.6%)			
Follow-up (years)	Median (IQ)	2.5 (1.5–3.7)			
Biochemical recurrence	37 (5.4%)				

Backward stepwise likelihood ratio method based on pre- and postoperative information reduced the number of predicting variables from 11 to 6 (Table 3). Histopathology grade group 3+, tumour size in histopathology and ADC were statistically significant. The LOOCV method also reduced the number of predicting variables to 6, where 4 of the 6 variables from Table 3 were the same for both methods.

**Table 3:** Predictors of early BCR by Cox hazards stepwise backward likelihood ratio

		*P* values		
		Unadjusted	Adjusted	Final model
Clinical + biopsy	Palpable tumour on DRE	0.0418*	0.878	
	% positive biopsies	0.0030*	0.9476	
	Age at operation	0.884	0.8304	
mpMRI	Radiological EPE present	1.8e^−06^*	0.0703	0.055†
	ADC value	2.95e^−05^*	0.0027*	0.001*
	T2W size on mpMRI (mm)	0.0018*	0.0545	0.074†
Histopathology	s-PSA within 3 days of operation	0.0123*	0.2148	
	Tumour size (ml)	3.97e^−06^*	0.0732	0.079†
	Grade group 3+	4.04e^−06^*	0.0001*	1.9e^−04^*
	Histology EPE present (Yes/No)	0.0006*	0.7786	
	Pos. margins (Yes/No)	0.0002*	0.1023	0.137†

*DRE* Digital rectal examination, *EPE* extra prostatic extension, *ADC* apparent diffusion coefficient, *T2W* T2-weighted

* *p* < 0.05 (two-sided). † Tumor size, radiological EPE and surgical margins were retained in the final model despite *p* > 0.05

**Fig. 2:**
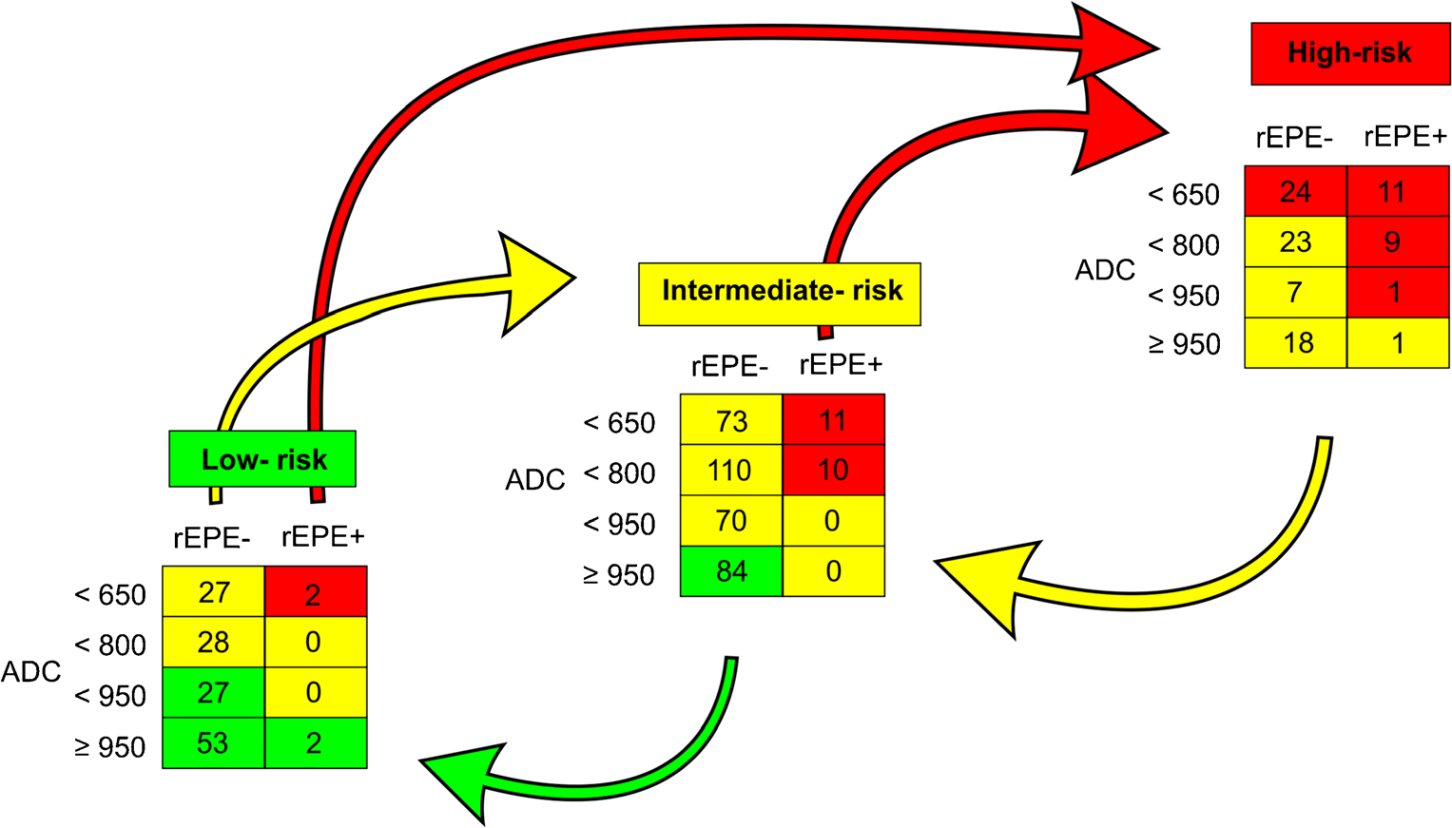
Reallocation of patients within CAPRA by using image adjustment. Illustrating the number of patients in each category and effect (by colour/arrows) of application of image adjustment (IA) to one of the two major risk stratification tools, CAPRA. Numbers in squares are the numbers of patients in each category given for CAPRA

Predictions by both the risk stratification tools and respective IA models and outcomes are summarised in Table 4. For the CAPRA model, image adjustment moved 55 (39.6%) of the low-risk patients into the intermediate-risk group and 2 (1.4%) into the high-risk group. The IA model further moved 84 (23.5%) of the intermediate-risk patients into the low-risk group and 21 (5.9%) into the high-risk group. In the CAPRA high-risk group, the IA model moved 49 patients (52.1%) down to the intermediate-risk group. Similar results were found for IA–D’Amico (data not shown). The crude numbers and effect of application of the IA model to CAPRA are shown in Fig. 2.

**Table 4:** Distribution of biochemical recurrence in the preoperative risk stratification models

	Low risk		Intermediate risk		High risk	
	BCR/total		BCR/total		BCR/total	
CAPRA	7/139	5.0%	14/358	3.9%	16/94	17.0%
IA–CAPRA	2/163	1.2%	20/360	5.6%	15/68	22.1%
D’Amico	7/126	5.6%	18/370	4.9%	12/95	12.6%
IA–D’Amico	2/159	1.3%	14/366	3.8%	14/64	21.8%

Despite allocating extra patients to the low-risk group, BCR risk in the low-risk group decreased from 4.5–5% to 1.1% for both models. Furthermore, both IA models reduced the number of high-risk patients with a concomitant increase in the BCR risk in the remaining high-risk patients.

**Fig. 3:**
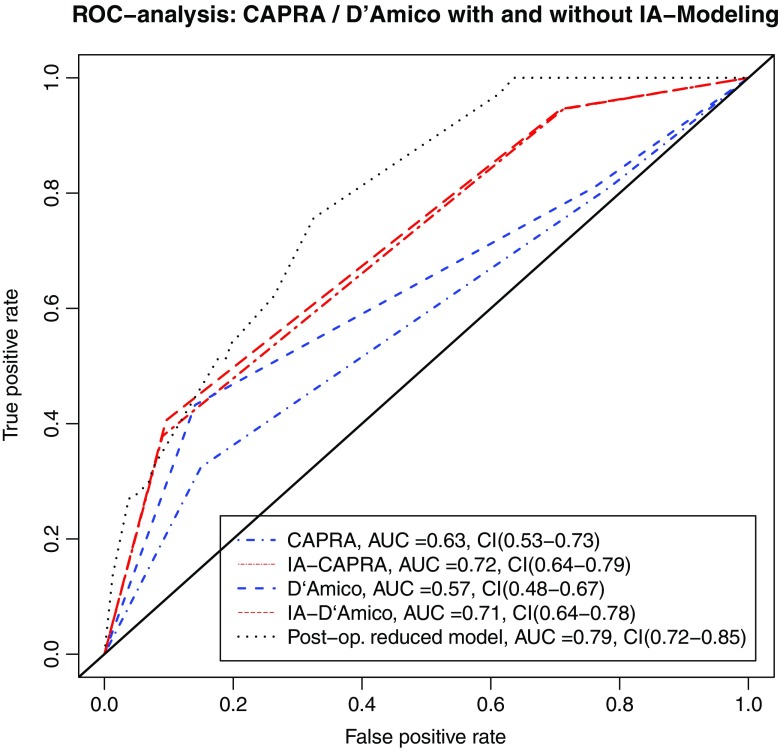
Receiver operating characteristics (ROC) comparing original risk stratification tools and the IA models. ROC curves of the two major risk stratification tools, CAPRA and D’Amico, with and without image adjustment; IA–CAPRA and IA–D’Amico, including area under the curve (AUC) and confidence intervals (CI). The reduced postoperative model includes all risk predictors including postoperative histopathology (see text for details)

**Fig. 4:**
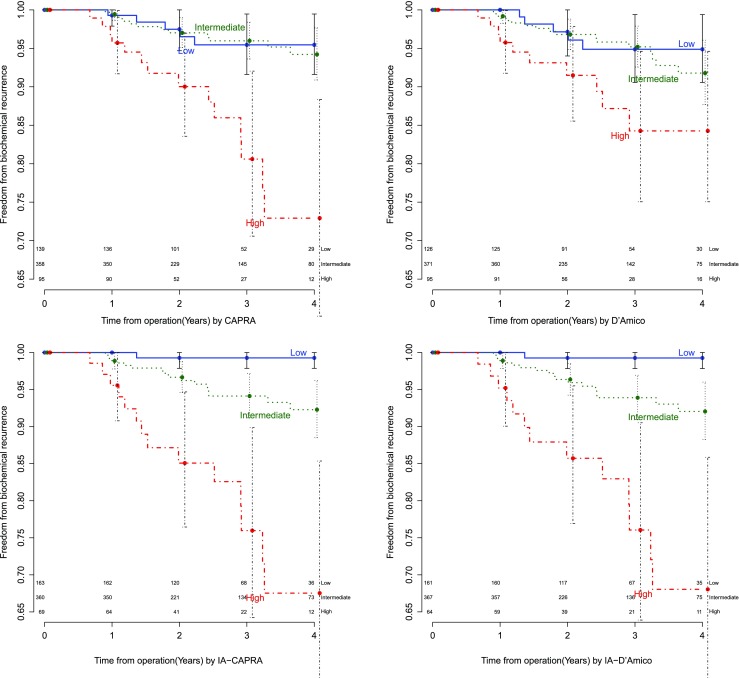
Kaplan–Meier curves illustrating risk stratification of biochemical recurrence (BCR) during the first 4 years of follow-up after prostatectomy for the two major risk stratification tools, CAPRA and D’Amico, with and without image adjustment

**Fig. 5:**
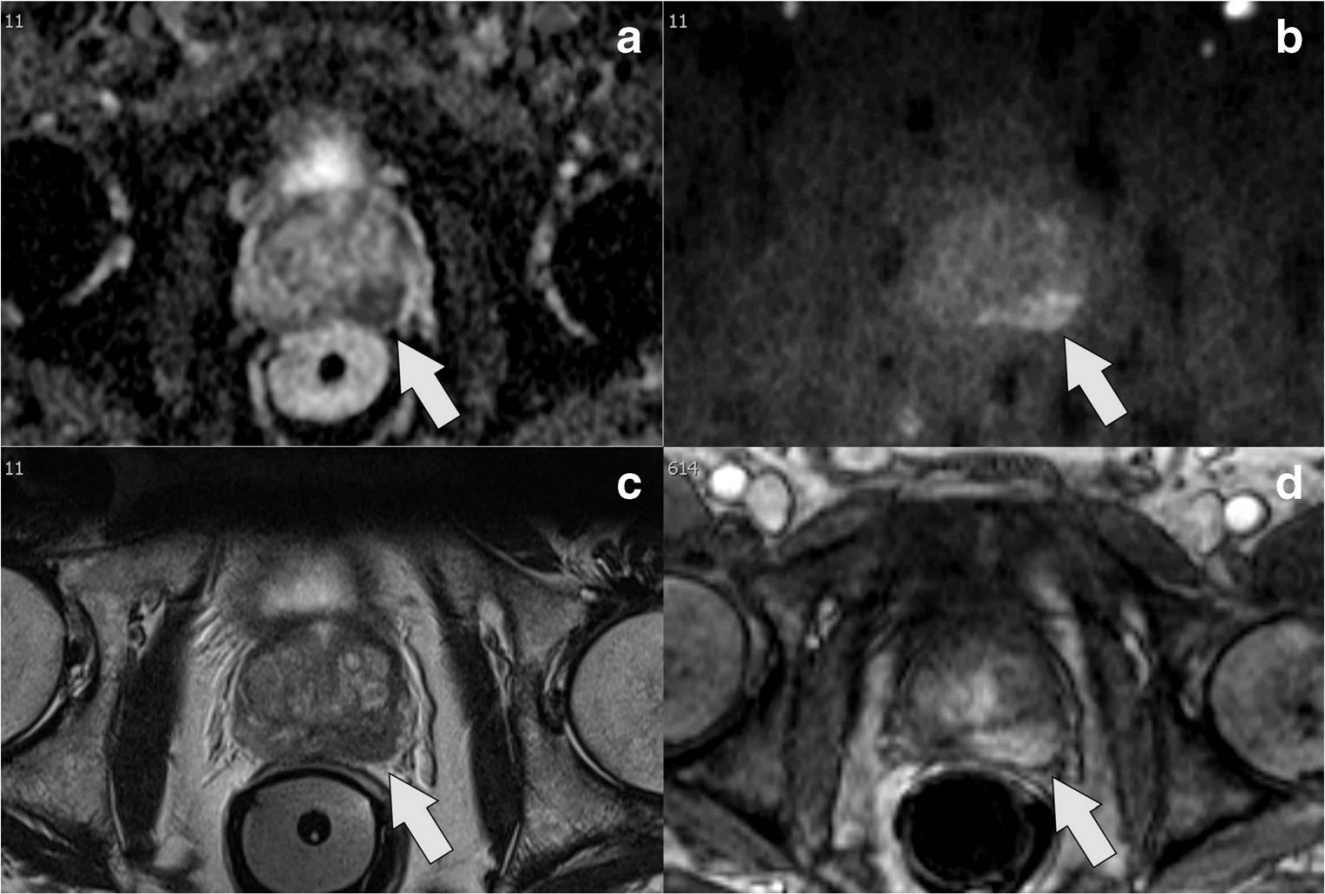
Example of risk increase (low to high) by IA models: a ADC map, b high *b* value (b1200), c T2W image, d dynamic contrast-enhanced; clinical information: s-PSA, 4.6; biopsy Gleason grade, 3 + 4; 25% pos. cores; CAPRA score = 2/D’Amico = intermediate. MRI shows significant tumour on the left side, ADC value = 629 mm^2^/s and rEPE+, Likert score = 4. Image adjusted to high risk. Histopathology after prostatectomy, pT3a; EPE = 4 mm; tumour volume = 1.5 ml; Gleason grade, 3 + 4; neg. margins, neg. lymph nodes; BCR within 1 year after operation

**Fig. 6:**
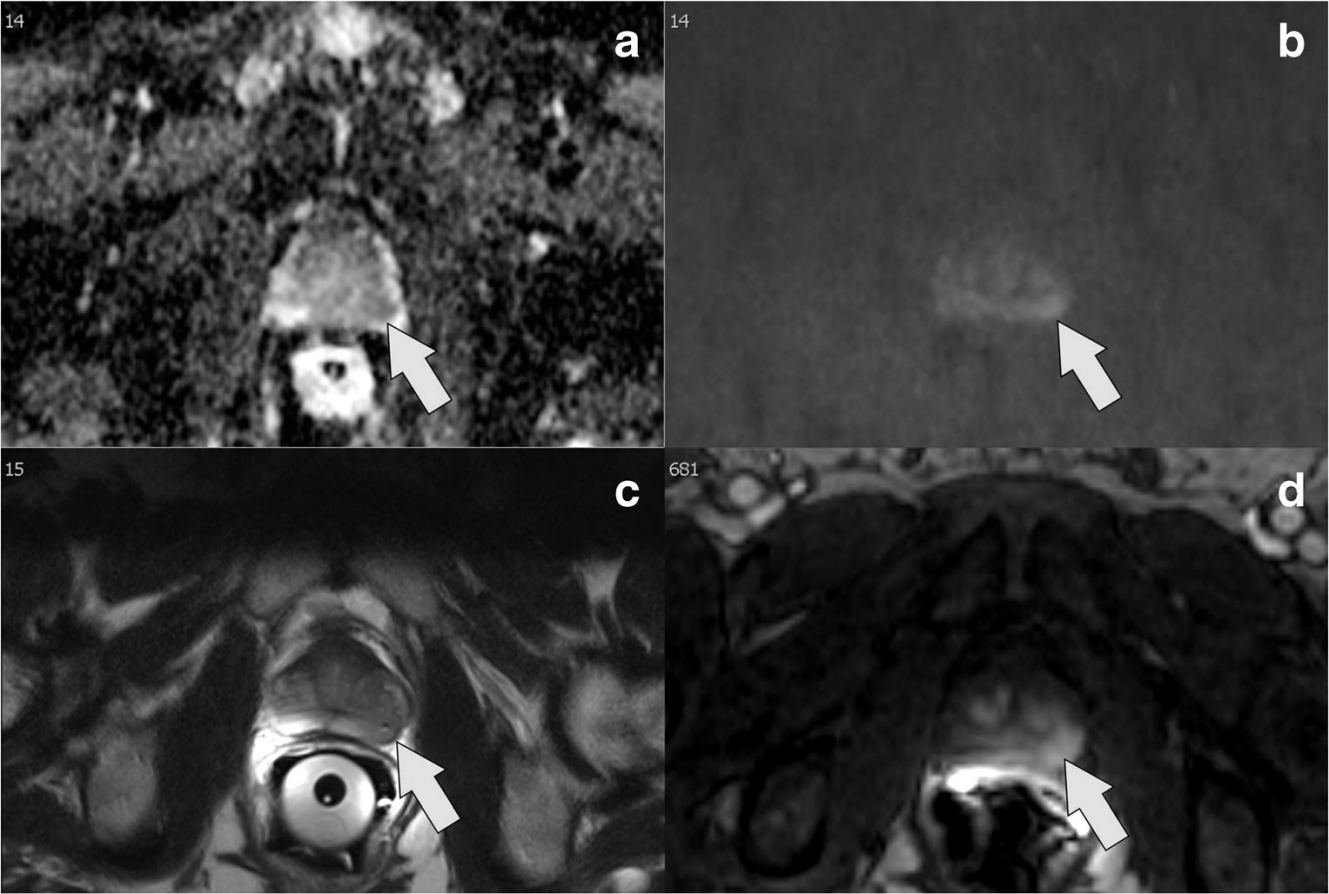
Example of risk reduction (high to intermediate) by IA models: a ADC map, b high *b* value (b1200), c T2W image, d dynamic contrast-enhanced; clinical information: s-PSA, 9.8; biopsy Gleason grade, 4 + 5; 90% pos. cores; CAPRA score = 6, D’Amico = high risk. MRI demonstrates mildly restricted diffusion on DWI, ADC value = 1006 mm^2^/s and rEPE–, Likert score = 3. Image adjusted to intermediate risk. Histopathology after prostatectomy, pT3a; EPE = 0.5 mm; tumour volume = 3.9 ml; Gleason grade, 3 + 4; neg. margins; no BCR within 3 years after operation

ROC analysis (Fig. 3) as well as the supplementary decision curve analysis (Fig. S3) confirmed that both IA models improved prediction of BCR over the well-established stratification tools. IA–CAPRA outperformed the other preoperative risk assessment tools as assessed by ANOVA and the IA models had significantly improved AUC, compared to the model they arrived from (*p* = 0.017, IA–CAPRA, and *p* = 0.008 IA–D’Amico). However, the postoperative reduced model which includes histopathology and surgical margins was still best (Fig. 3 and Fig. S3). Kaplan–Meier curves for disease-free survival (Fig. 4) show a better discrimination between the three risk groups for both IA–CAPRA and IA–D’Amico as compared to the original risk stratification tools. Examples of increased and decreased risk after image adjustment are given in Figs. 5 and 6.

## Discussion

The present study represents a new approach to integrating imaging features into preoperative risk stratification tools for risk of recurrence. Instead of developing completely new risk prediction tools predominantly based on imaging information, we sought to retain the predictive power of two well-established preoperative risk prediction tools for PCa, adding information from pertinent imaging features weighted according to their relative risk contribution. Our approach is essentially more conservative than setting up a completely new model and helps to avoid overfitting the prediction models to a given institutional cohort. This methodology could also be applied to other cancers where imaging is valuable e.g. breast cancer and rectal cancer [31, 32]. Dependable pretreatment risk assessment is essential to select the most appropriate treatment, such as radical therapy for a patient with PCa.

This is a large retrospective cohort study showing a clear benefit of integrating mpMRI features into well-established preoperative risk stratification tools, CAPRA and D’Amico, to predict risk of BCR for PCa. In the 2016 revision of the European Association of Urology (EAU) guidelines, mpMRI was endorsed as a tool for assessing clinical tumour (cT) stage in high-risk patients [1]. To our knowledge, the effect of this new recommendation on the preoperative risk predictions has not yet been validated. As an alternative approach to improving preoperative risk stratification, we investigated the impact of mpMRI features on risk stratification without altering the basis for cT stage i.e. clinical findings on DRE. We decided not to study the EAU criteria since utilising image information to override cT stage would result in a T stage migration, which would be difficult to adjust for when evaluating the effect of integrating mpMRI features into new prediction tools. The usefulness of including preoperative information in addition to postoperative information—until now the gold standard for risk prediction—has been recently shown by Imnadze et al. [33].

Zhang et al. [17], using an MRI nomogram to predict BCR, identified that patients with a low ADC, high MRI T stage and high PI-RADS score had significantly higher risk of BCR by univariate survival analysis. Further, given that ADC and PI-RADS score were significant predictors of BCR in multivariate Cox regression analysis, these findings are in line with our study. Ho et al. [18] identified both mpMRI suspicion score (low, moderate, high) and rEPE as predictors of BCR together with Gleason score. The finding of rEPE and Gleason score as independent predictors is in line with our study, but their mpMRI suspicion score is related to the number of sequences suggestive of cancer and is thus difficult to compare with the ADC-threshold in our study.

Using our proposed IA models increased the number of patients in the low-risk groups by 6 percentage points. At the same time, risk of BCR in the low-risk groups was reduced from approx. 5% to 1.1% using both IA prediction tools. This could potentially reduce the number of patients selected for radical treatment, offering them the alternative treatment strategy of active surveillance (AS). This potential approach should ideally be evaluated in a prospective randomised non-inferiority trial based on IA–CAPRA comparing outcomes between AS and radical treatment in the low-risk group. The IA–CAPRA model also reduced the number of patients in the high-risk group by 52% with a concomitant increase in risk of BCR in the remaining patients from 17% to 22%, leading to more specific predictions also at the high-risk end of the spectrum.

The differences between IA–CAPRA and IA–D’Amico were marginal and performance almost equal, except for the ANOVA analysis alone. Not all of the well-established nomograms can be applied to any population [34]. If general performance of a prognostic tool is low, this will pull down the performance of the IA model.

The presented IA model is basically conservative and depends on the primary risk stratification tools being used. The mpMRI features, when applied, will only upgrade a low-risk patient to the high-risk group if there is a huge mismatch between mpMRI and preoperative clinical risk assessment. It is worth noting that in our presented IA models, mpMRI features including quantitative measurements of ADC can be used to both upgrade or downgrade risk in a given patient, in line with previous publications [35, 36]. Studies using qualitative PI-RADS criteria to predict postoperative Gleason scores have similarly shown image-based up- and downgrading [37, 38]. In a recent study by Park et al. [16] utilising PI-RADS to predict BCR in a highly selected series of 158 patients followed up for 2 years after operation at a single institution, PI-RADS score of at least 4 indicated higher risk of BCR in multivariate analysis. In contrast to Park et al., our study employed a larger and more homogeneous cohort, quantitative criteria for ADC, and LOOCV to assess the relative weighting of potential mpMRI predictive features.

A potential objection to the IA model may be that a clinically or bioptically missed significant tumour will only be partially corrected by the proposed IA model. In cases with a significant mismatch between radiology and biopsies, repeat biopsy under image guidance should be carefully considered [39].

The present study has the following limitations. (i) Our study was conducted at a single institution with a limited number of patients. (ii) The observed frequency of BCR of 6.3% is lower than reported in previous studies [10, 11, 16–18]. This may partially be explained by our careful exclusion of patients with persistent disease. (iii) The proportion of patients with high risk in our cohort is lower than in other series. However, the selection of patients was in accordance with the guidelines at that time, and the results regarding surgical margins were within the expected range. (iv) Differences between scanners and imaging techniques will probably result in slightly different cut-off points, presumably in a similar range around 650, 800 and 950 mm^2^/s. However, Yoon et al. [19] dichotomised at the ADC level of 746 mm^2^/s. The differences in ADC cut-off from our study were most probably due to differences in scanner, *b* values and their high-risk patient cohort. Zhang et al. [17] dichotomised at 950 mm^2^/s, with no information regarding why they chose such a threshold.

Differences in ADC measurements between scanners can be adjusted for with calibration using quantitative phantoms. Increasing our patient cohort to patients scanned in multiple scanners (*n* = 680) had no detectable effect on our IA model results (analyses not shown). (v) Our model needs to be externally validated in an independent cohort. Special consideration was paid to statistical methods for estimates of predictors with close attention to advice from expert biostatisticians.

In conclusion, integrating information from mpMRI of the prostate into well-established clinical risk stratification tools for BCR, CAPRA and D'Amico allows better predictions of BCR and a better differentiation between risk groups, facilitating a more appropriate choice of follow-up at the different risk levels. The improved risk stratification by mpMRI could potentially result in a more appropriate choice of therapy i.e. active surveillance versus radical therapy.

## Electronic supplementary material

ESM 1 (EPS 4.77 kb)

ESM 2 (EPS 545 kb)

ESM 3 (EPS 9.72 kb)

## Abbreviations

ADC: apparent diffusion coefficient
AIC: Akaike information criterion
BCR: biochemical recurrence
CAPRA: University of California San Francisco Cancer of the Prostate Risk Assessment tool
cT: clinical T stage
EPE: extraprostatic extension
IA: image-adjusted
ISUP: International Society of Urological Pathology
LOOCV: leave-one-out cross-validation
mpMRI: multiparametric MRI
PCa: prostate cancer
RALP: robot-assisted laparoscopic prostatectomy
RP: radical prostatectomy
rEPE: radiological EPE
SM+: positive surgical margins
